# Diversification of blaOXA-48-harbouring plasmids among carbapenemase-producing Enterobacterales, 11 years after a large outbreak in a general hospital in the Netherlands

**DOI:** 10.1099/mgen.0.001335

**Published:** 2025-01-10

**Authors:** Pieter W. Smit, Carla van Tienen, Fabian Landman, Sabrina Zagers, Marije den Drijver, Arjan Burggraaf, Daan W. Notermans, Marjolein Damen, Antoni P.A. Hendrickx, Casper Jamin

**Affiliations:** 1 Maasstad Laboratories, Medical Microbiology, Molecular Diagnostics Unit, Maasstad Hospital, Rotterdam, Netherlands; 2 Center for Infectious Disease Control (CIb), National Institute for Public Health and the Environment (RIVM), Bilthoven, Netherlands

**Keywords:** *bla_OXA-48_*, *Enterobacterales*, outbreak, transmission

## Abstract

**Introduction.** Genes encoding OXA-48-like carbapenem-hydrolyzing enzymes are often located on plasmids and are abundant among carbapenemase-producing *Enterobacterales* (CPE) worldwide. After a large *bla*_OXA-48_ plasmid-mediated outbreak in 2011, routine screening of patients at risk of CPE carriage on admission and every 7 days during hospitalization was implemented in a large hospital in the Netherlands. The objective of this study was to investigate the dynamics of the hospitals’ 2011 outbreak-associated *bla*_OXA-48_ plasmid among CPE collected from 2011 to 2021.

**Methods.** A selection of 86 *bla*_OXA-48_-carrying CPE isolates was made from 374 isolates collected over an 11-year study period. Species included *Escherichia coli* (Eco), *Klebsiella pneumoniae* (Kpn), *Enterobacter cloacae complex* (Ecl), *Citrobacter freundii* (Cfr), *Citrobacter koseri* (Cko) and *Morganella morgani* (Mmo). Short-read sequencing was combined with long-read sequencing for all isolates to reconstruct *bla*_OXA-48_-like plasmids and chromosomes of CPE. MASH, MOBsuite, ResFinder, PlasmidFinder and SNP analyses were performed to study diversity. pOXA-48 plasmids were compared to plasmid sequences that were sequenced for the Dutch CPE surveillance in the same time period.

**Results.** In total for the 86 CPE, 2 failed genomic assemblies and 78 *bla*_OXA-48_-encoding plasmids were reconstructed, and six *bla*_OXA-48_ genes were located chromosomally. The 2011 outbreak-associated *bla*_OXA-48_ plasmid of 63.6 kb with IncL replicon was found in Cfr, Ecl, Eco, Kpn and Mmo and primarily between 2011 and 2014 and indicated as LR025105 as MASH nearest neighbour. From 2014 onwards, 11 other types of *bla*_OXA-48_-carrying plasmids with different antibiotic-resistant genes and replicons were discovered, representing the earlier defined distinct pOXA-48 plasmid groups found in the Netherlands. Furthermore, on a national level, the LR025105 plasmid was found after 2015 in many different bacterial backgrounds, highlighting the promiscuous nature of this pOXA-48 plasmid.

**Conclusion.** After a large *bla*_OXA-48_ outbreak in a large hospital in the Netherlands, the composition of the *bla*_OXA-48_ plasmid population in this hospital diversified over time and is in line with national surveillance data. Plasmid sequencing provided valuable insight into the transmission dynamics of *bla*_OXA-48_-encoding plasmids and showed no indication of the persistence of the 2011 *bla*_OXA-48_ plasmid in the hospital environment.

­

**Impact Statement**

OXA-48 carbapenem-hydrolyzing enzymes encoded from plasmids or chromosomes of *Enterobacterales* have spread globally and are of concern in the hospital setting. This is one of the first studies to investigate the diversification and transmission dynamics of *bla*_OXA-48_-carrying plasmids in a large hospital in comparison to the national surveillance of *bla*_OXA-48_-carrying plasmids over an 11-year study period, highlighting the need for surveillance of not only bacterial strains but also antimicrobial resistance-encoding mobile genetic elements.

## Data Summary

The plasmid and chromosome sequences are deposited in GenBank of the National Center for Biotechnology Information and available through the accession numbers PRJNA1116077, PRJEB42331, PRJNA691727 and PRJEB35685. PRJNA1116077: Diversification of blaOXA-48-harbouring plasmids among carbapenemase-producing *Enterobacterales*, 11 years after a large outbreak in a general hospital in the Netherlands with 85 biosamples and 84 assemblies. PRJEB42331: blaOXA-48-like genome architecture among carbapenemase-producing *Escherichia coli* and *Klebsiella pneumoniae* in the Netherlands with 167 biosamples and 167 SRA experiments. PRJNA691727: blaOXA-48-like genome architecture among carbapenemase-producing *E. coli* and *K. pneumoniae* in the Netherlands with 223 biosamples and 44 assemblies. PRJEB35685: Molecular characteristics of Carbapenemase-producing Enterobacterales in the Netherlands; results of the 2014–2018 national laboratory surveillance with 891 biosamples and 891 SRA experiments. The authors confirm that all supporting data and accession numbers have been provided within the article and through supplementary data files.

## Introduction

Genes encoding OXA-48-type carbapenemase enzymes have disseminated among the family of *Enterobacterales* and represent a major concern globally for patient care and public health [1]. In carbapenemase-producing *Enterobacterales* (CPE), OXA-48-type carbapenemases are typically located on conjugative plasmids that can transfer intra- and interspecies, conferring resistance to carbapenem antibiotics [2,3]. Most hospital infection control measures are based on tracking the transmission of the same antimicrobial-resistant bacterial species, thereby potentially missing plasmid-based outbreaks among multiple different CPE [1,2].

In a large hospital in the Netherlands in late 2010, a *Klebsiella pneumoniae* with multilocus sequence typing (MLST) sequence type ST395 and later *Escherichia coli* ST88 carrying a *bla*_OXA-48_ plasmid caused a large outbreak, having a major effect on both patients and hospital staff [3]. The *bla*_OXA-48_ harbouring *K. pneumoniae* outbreak-associated isolates were phenotypically resistant to meropenem and imipenem, whereas most *E. coli* isolates carrying *bla*_OXA-48_ were phenotypically susceptible to these antibiotics [4]. The *bla*_OXA-48_ gene was embedded in a transposon Tn1999.2 and located on a 62 kb IncL/M conjugative plasmid, detected in 14 different species [5]. The spread of this plasmid was most likely caused by the European dissemination of a single *K. pneumoniae* clone with ST395 [6]. While this was probably the first *bla*_OXA-48_ plasmid-associated hospital outbreak in the Netherlands, it did not result in widespread dissemination of one plasmid in the Dutch population as the genetic diversity of *bla*_OXA-48_ carrying plasmids became high [7]. After the outbreak (2014–2018), the CPE population in the Netherlands was diverse with varying carbapenemase alleles, suggesting multiple introductions into the country [8].

To tackle the hospital outbreak, a carbapenemase gene PCR-based screening method was developed and implemented in 2011, facilitating the detection of cross-transmission and horizontal gene transfer of *bla*_OXA-48_ [9]. To prevent potential future outbreaks, rigid antimicrobial resistance surveillance remained after the outbreak, screening on average 4000 patients a year. The hospital-based antimicrobial resistance surveillance therefore provides a unique insight into the transmission of resistance genes at the hospital level over a long period of time. Because of the unique setting in which many patients are routinely screened for CPE, we set out to investigate the genomic epidemiology of *bla*_OXA-48_ in a hospital setting over an 11-year study period. The objectives of this study were to (1) determine the temporal dynamics of different *bla*_OXA-48_ plasmids in a large hospital between 2011 and 2021, using short- and long-read sequencing, and (2) to compare the epidemiology of the outbreak-associated *bla*_OXA-48_ plasmid to those obtained from CPE submitted to the Dutch national CPE surveillance programme.

## Methods

### Clinical setting

This study was performed in a 600-bed hospital located in Rotterdam, the second largest city in the Netherlands. The hospital where this study took place has ~27,000 admissions and 138,000 outpatient clinic visits each year.

During the initial outbreak, patient screening and surveillance were intensified, as screening for Methicillin Resistant Staphylococcus Aureus (MRSA) in nationally defined risk groups was already in place before 2004. The following patients were screened for MRSA and multidrug-resistant Gram-negative organisms upon admittance to the hospital since 2011 : (1) those who were hospitalized in a foreign country 2 months prior to being hospitalized in a Dutch hospital; (2) when admitted to the neonatal intensive care unit (ICU), ICU or burn centre; (3) all patients at every seventh day of stay within the hospital; and (4) patients at risk of MRSA carriage based on a nationally standardized questionnaire. In addition, patients with selective decontamination of the digestive tract on the ICU were screened biweekly [10]. Furthermore, when transmission was suspected, transmission investigations were conducted.

From the screening samples collected in the study period, a selection of CPE isolates was made, representing the diversity of CPE isolates longitudinally over the 11-year period. No formal randomization procedure was used, but 86 isolates were selected aiming to maximize diversity (species, patient and time) with limited long-read sequencing capacity due to financial constraints. This was done to prevent overrepresentation of outbreak-related intensified screening samples. The sample selection was further based on CPE species, patient ID and date, thus blinded to any patient details, hospitalization details or any infection prevention information.

### National surveillance of CPE

Medical microbiology laboratories in the Netherlands are invited to send *Enterobacterales*, *Pseudomonas aeruginosa* and *Acinetobacter baumannii* complex, suspected of carbapenemase production to the National Institute of Public Health and Environment (RIVM), as part of national surveillance [8,11]. As the incidence of CPE carriage and infections remained sporadic, outside the 2011 *bla*_OXA-48_ outbreak, routine and structured national surveillance by short- and long-read sequencing for CPE started from 2016 onwards.

### Molecular screening

A PCR-based screening method was developed and implemented, facilitating the detection of cross-transmission and horizontal gene transfer of the *bla*_OXA-48_ gene [9]. In brief, PCRs targeting *bla*_VIM_, *bla*_NDM_, *bla*_OXA-48-like_, *bla*_KPC_, *bla*_GES_ (since 2018) and *bla*_IMP_ were performed on 24-h cultured rectal swabs, as described previously [9]. If one of the PCRs was positive, overnight broth was cultured to perform species identification using MALDI-TOF (Vitek, bioMérieux) and to determine phenotypical resistance.

### Next-generation sequencing

CPE isolates were subjected to next-generation sequencing (NGS) using Illumina MiSeq V2 (Illumina, USA). All bioinformatic tools were run using default parameters unless otherwise specified. The antibiotic-resistant gene profile and plasmid replicon compositions were determined by interrogating the ResFinder (version 4.1), PlasmidFinder (version 2.0.1) databases available from the Center for Genomic Epidemiology and MOBsuite (version 3.0). For ResFinder, a 95% identity threshold and a minimum length of 60% were used as criteria, whereas for PlasmidFinder, an identity of 95% was utilized. The resulting NGS-derived data, such as resistance genes, replicons, wgMLST profiles and MOBsuite, were imported into BioNumerics version 7.6.3 for subsequent comparative analyses (Applied Maths, Sint-Martens-Latem, Belgium), as described previously [7].

### Nanopore long-read sequencing

High-molecular-weight DNA was isolated using an in-house developed protocol as described previously [7]. The Oxford Nanopore rapid barcoding kit (SQK-RBK004) and protocol (https://community.nanoporetech.com) were used (Oxford Nanopore Technologies, Oxford, UK). Barcoded isolates were pooled, and sequencing adapters were attached. The final library was loaded onto a MinION flow cell (MIN-106 R9.4.1). The 48-h sequence run was started with live base calling enabled using the MinKNOW software on a GridION device. After the sequence run, de-multiplexing was performed using Guppy, resulting in a single FASTQ file per isolate. Eighty base pairs were trimmed at both sides, and reads larger than 5000 and the 90% best scoring reads were used in further analyses using NanoFilt (v2.8.0) and Filtlong (v0.2.1). Illumina and Nanopore data were used in a hybrid assembly performed by Unicycler (v0.4.4) [12]. Illumina data were not trimmed before running Unicycler, which was operated using default settings and verbosity 2. The resulting contig files were annotated using Prokka (v1.14.6) [13] and were subsequently loaded into BioNumerics for further analyses. The genetic context was visualized with SnapGene viewer.

### Plasmid and chromosome comparisons

For comparative purposes, plasmid groups were defined using MASH nearest neighbours by MOBsuite. BioNumerics was used to compare complete plasmid DNA sequence and chromosome datasets. Linear assembly contigs were omitted in this study. CLC Genomics Workbench software (v12, www.qiagenbioinformatics.com) was used to retrieve *bla*_OXA-48_-like plasmids and chromosomes from previously sequenced CPE from the Netherlands. These plasmids and chromosomes were stripped from their annotations and re-annotated again using Prokka v1.14.6. The number of SNPs between plasmids was determined using SKA (v1.0).

## Results

### Presence of *bla*_OXA-48_-carrying CPE in a large hospital, 2011–2021

**Fig. 1.**
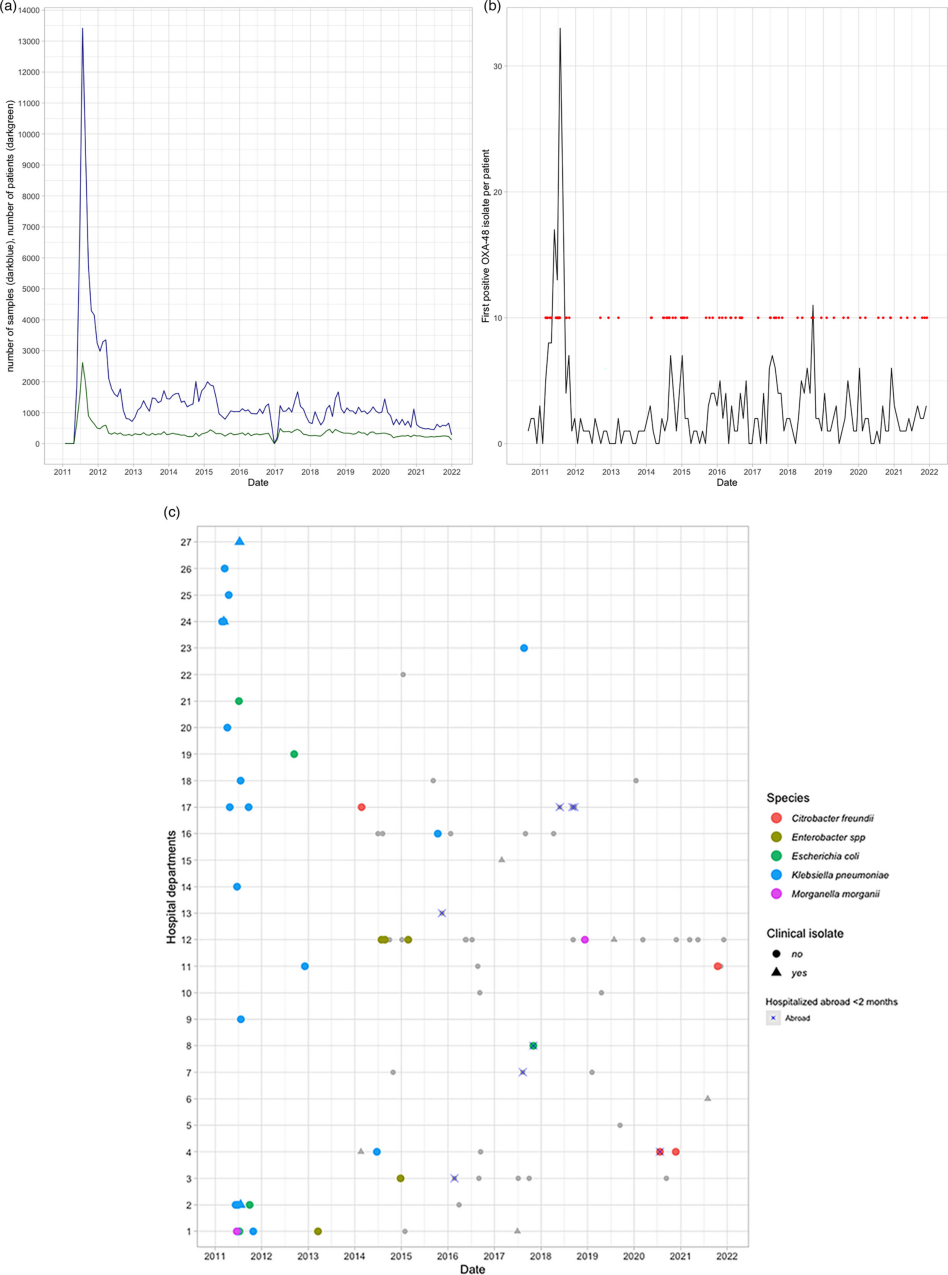
Screening for *bla*_OXA-48_ positivity at a Dutch hospital, 2011–2021. (**a**) Number of patients screened (green) and samples taken (blue) in the study from 2011 to 2021. (**b**) Number of *bla*_OXA-48_-positive CPE isolates over an 11-year period. Number of *bla*_OXA-48_-positive isolates (*n*=374) found from both routine screening and clinical samples, with the first positive isolate per patient counted, during 1 January 2011 until 31 December 2021. The red dots indicate the sampling moment of the 86 samples that were selected for sequencing. (**c**) Plasmids with MASH nearest neighbour LR025105 were plotted based on sampling date, type of organism and whether the patient had visited a foreign hospital in the last 2 months (>24 h stay) with non-LR025105 plasmids shown in grey. Those samples plotted with an ‘x’ are patients who were screened and found *bla*_OXA-48_ culture positive upon arrival due to hospitalization abroad. A triangular shape indicates that isolates were obtained from a clinical site (e.g. urine, sputum, wound and blood) and a round shape indicates it was not (rectum swab and faeces).

In the 11-year study period, ~187,000 samples from 47,200 patients were screened for CPE from 2011 to 2021, including *bla*_OXA-48_-carrying CPE (Fig. 1a). The largest number of *bla*_OXA-48_-positive CPE was detected in 2011 during the outbreak. In the years that followed, *bla*_OXA-48_-carrying CPE were detected intermittently throughout the 11-year study period, with a large decline in recorded samples tested in January 2017, as records of samples tested between December 2016 and February 2017 got lost.

Out of the 47,200 patients, 374 (0.8 %) were carriers of *bla*_OXA-48_-like-positive CPE when screened for carbapenemase genes (Fig. 1b). A cross-sectional selection of isolates was made (screening clinical samples and counting positive isolate per patient first) to closely represent the diversity of isolates longitudinally over the 11-year period (Fig. 1b). In total, 86 isolates were selected, taken from the freezers, cultured and sequenced with both short-read and long-read sequencers. Two of the 86 samples did not contain a *bla*_OXA-48_ gene, and one sample failed the hybrid assembly. Both were, therefore, excluded from further analysis (*n*=84 samples).

**Fig. 2.**
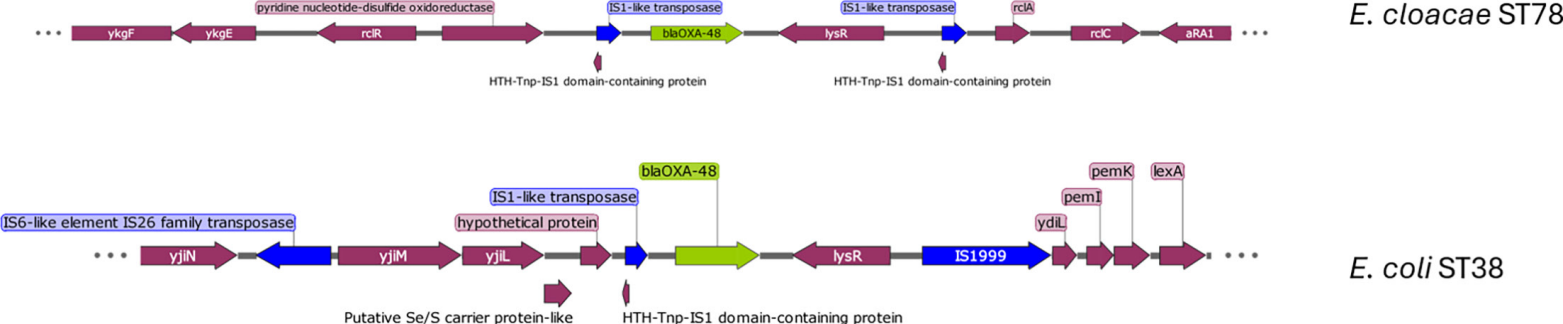
Layout of chromosomal bla_OXA-48_ isolates. Only 10 kb around the bla_OXA-48_ gene was shown to improve the readability of the figure. Only one of the chromosomal *E. coli* was shown to avoid redundancy.

The 84 successfully sequenced isolates comprised 31 *K. pneumoniae* (37%), of which 19 belonged to ST395, the first European-disseminated *bla*_OXA-48_ *K. pneumoniae*, 27 *Enterobacter* spp. (32%), 18 *E. coli* (21%), 5 *Citrobacter freundii* (6%), 2 *Morganella morganii* (2%) and 1 *Citrobacter koseri* (1%) (Table 1)*.* Out of the 84 isolates, for 78 (93%), the *bla*_OXA-48_ gene was located on plasmids, while for 6 (7%) isolates, *bla*_OXA-48_ was found chromosomally (Table 2). These six chromosomal *bla*_OXA-48_ isolates consisted of four ST38 *E. coli*, a known chromosomal *bla*_OXA-48_ carrier (10), and one ST78 *Enterobacter cloacae* (Fig. 2). The genetic context around the *bla*_OXA-48_ gene was identical among the five ST38. The first of these ST38 *E. coli* was found in 2014. These *E. coli* had three different transposases in the vicinity of the *bla*_OXA-48_ gene: IS6-like IS26 family tranposase and IS1999. The *E. cloacae* only had two IS1-like transposases flanking the *bla*_OXA-48_ and *LysR* gene, which suggests that these transposases formed a transposon with the AMR gene and moved these genes into the bacterial chromosome.

**Table 1.** Overview of CPE and sample type included in this study. ST, sequence type. nd stands for not determinable, either due to the absence of a suitable MLST scheme or due to the inability to call all seven loci to determine a sequence type. For *Citrobacter koseri*, a new ST was identified as this MLST scheme used is specifically for *Citrobacter freundii* species complex, to which *C. koseri* does not belong, yet still all *C. freundii* loci are present in this particular *C. koseri*. The new ST for *E. coli* was due to a novel variant in the fumC locus, with the nearest neighbour being fumC 40, leading to a novel ST, with ST1385 being the nearest neighbour

Overview of organisms, MLST types and sample types included, Dutch hospital 2011-2021									
MLST sequence type		Swab	Urine	Wound	Blood	Sputum	Faeces	Uknown	Total
*Citrobacter freundii*									
	ST111	1	0	0	0	0	0	0	1
	ST146	1	0	0	0	0	0	0	1
	ST22	1	0	0	0	0	0	0	1
	ST579	1	0	0	0	0	0	0	1
	ST91	1	0	0	0	0	0	0	1
*Citrobacter koseri*									
	New ST	1	0	0	0	0	0	0	1
*Enterobacter* spp.									
	nd	1	0	0	0	0	0	0	1
	ST114	1	0	0	0	0	0	0	1
	ST121	5	1	1	0	0	0	0	7
	ST78	10	1	2	1	2	0	2	18
*Escherichia coli*									
	New ST	0	0	0	0	1	0	0	1
	ST10	1	0	0	0	0	0	0	1
	ST1049	1	0	0	0	0	0	0	1
	ST127	1	0	0	0	0	0	0	1
	ST141	0	0	0	0	0	0	1	1
	ST144	1	0	0	0	0	0	0	1
	ST162	1	0	0	0	0	0	0	1
	ST2608	0	0	0	0	0	0	1	1
	ST38	4	0	0	0	0	0	0	4
	ST453	2	0	0	0	0	0	0	2
	ST58	1	0	0	0	0	0	0	1
	ST648	1	0	0	0	0	0	0	1
	ST88	2	0	0	0	0	0	0	2
*Klebsiella pneumoniae*									
	ST101	2	0	1	0	0	0	0	3
	ST134	1	0	0	0	0	0	0	1
	ST147	2	0	0	1	0	0	0	3
	ST16	0	0	1	0	0	0	0	1
	ST25	1	0	1	0	0	0	0	2
	ST307	1	0	0	0	0	0	0	1
	ST395	10	3	2	0	0	1	3	19
	ST976	1	0	0	0	0	0	0	1
*Morganella morganii*									
	nd	2	0	0	0	0	0	0	2
Sum	—	58	5	8	2	3	1	7	84

**Table 2.** Overview of bla_OXA-48_ locations, plasmid types and predicted mobility of 84 isolates, based on MOBsuite analysis, sequenced from one hospital in the Netherlands, 2011-2021

Chromosomally or plasmid located OXA-48 genes detected, Dutch hospital 2011-2021							
Plasmid	Location	Type	Predicted mobility	% GC	length bp	n	%
*Citrobacter freundii*							
LR025105	Plasmid	IncL/M	Conjugative	51.22	63,589	2	2.4
KX636096	Plasmid	IncL/M	Conjugative	51.24	60,959	1	1.2
LR025105	Plasmid	IncL/M	Conjugative	51.18	63,497	1	1.2
LR025105	Plasmid	IncL/M	Conjugative	51.18	63,499	1	1.2
*Citrobacter koseri*							
KX636096	Plasmid	IncL/M	Conjugative	51.23	62,041	1	1.2
*Enterobacter*spp.							
LN864820	Plasmid	IncL/M	Non-mobilizable	51.48	49,887	12	14.3
KX636096	Plasmid	IncL/M	Conjugative	51.23	62,041	5	6.0
LR025105	Plasmid	IncL/M	Conjugative	51.22	63,589	4	4.8
	Chromosome			55.18	4,731,943	1	1.2
CP023251	Plasmid	IncL/M	Conjugative	51.19	62,812	1	1.2
KX636096	Plasmid	IncL/M	Conjugative	51.17	61,174	1	1.2
KX636096	Plasmid	IncL/M	Conjugative	51.19	61,988	1	1.2
KX636096	Plasmid	IncL/M	Conjugative	51.23	62,035	1	1.2
LR025105	Plasmid	IncL/M	Conjugative	51.21	63,589	1	1.2
*Escherichia coli*							
LR025105	Plasmid	IncL/M	Conjugative	51.21	63,589	2	2.4
LR025105	Plasmid	IncL/M	Conjugative	51.22	63,589	2	2.4
	Chromosome			50.44	5,185,030	1	1.2
	Chromosome			50.51	5,211,965	1	1.2
	Chromosome			50.6	5,321,360	1	1.2
	Chromosome			50.66	5,170,888	1	1.2
	Chromosome			50.66	5,265,106	1	1.2
CP017989	Plasmid	IncR	Mobilizable	51.9	86,140	1	1.2
CP023251	Plasmid	IncL/M	Conjugative	50.79	65 728	1	1.2
CP045282	Plasmid	IncFIA	Mobilizable	52.75	69,156	1	1.2
KX636096	Plasmid	IncL/M	Conjugative	51.23	62,041	1	1.2
KX636096	Plasmid	IncL/M	Conjugative	51.29	62,444	1	1.2
KX636096	Plasmid	IncL/M	Conjugative	52.29	78,593	1	1.2
KY200950	Plasmid	IncL/M	Non-mobilizable	53.12	32,829	1	1.2
LR025105	Plasmid	IncL/M	Conjugative	51.24	64,365	1	1.2
NC_019089	Plasmid	IncFIA, IncFIC	Conjugative	53.42	110,596	1	1.2
*Klebsiella pneumoniae*							
LR025105	Plasmid	IncL/M	Conjugative	51.22	63,589	20	23.8
CP018452	Plasmid	IncL/M	Conjugative	50.57	67,100	2	2.4
KX636096	Plasmid	IncL/M	Conjugative	52.29	78,593	2	2.4
CP018443	Plasmid	IncL/M	Non-mobilizable	51.47	49,863	1	1.2
CP027039	Plasmid	IncL/M	Conjugative	51.23	63,675	1	1.2
KX523901	Plasmid	IncL/M	Conjugative	51.44	66,276	1	1.2
KX636096	Plasmid	IncL/M	Conjugative	51.27	61,781	1	1.2
LR025105	Plasmid	IncL/M	Conjugative	51.22	63,578	1	1.2
LR025105	Plasmid	IncL/M	Conjugative	51.25	64,366	1	1.2
NC_019089	Plasmid	IncFIA,IncFIC	Conjugative	53.42	110,596	1	1.2
*Morganella morganii*							
LR025105	Plasmid	IncL/M	Conjugative	51.22	63,589	2	2.4

**Fig. 3.**
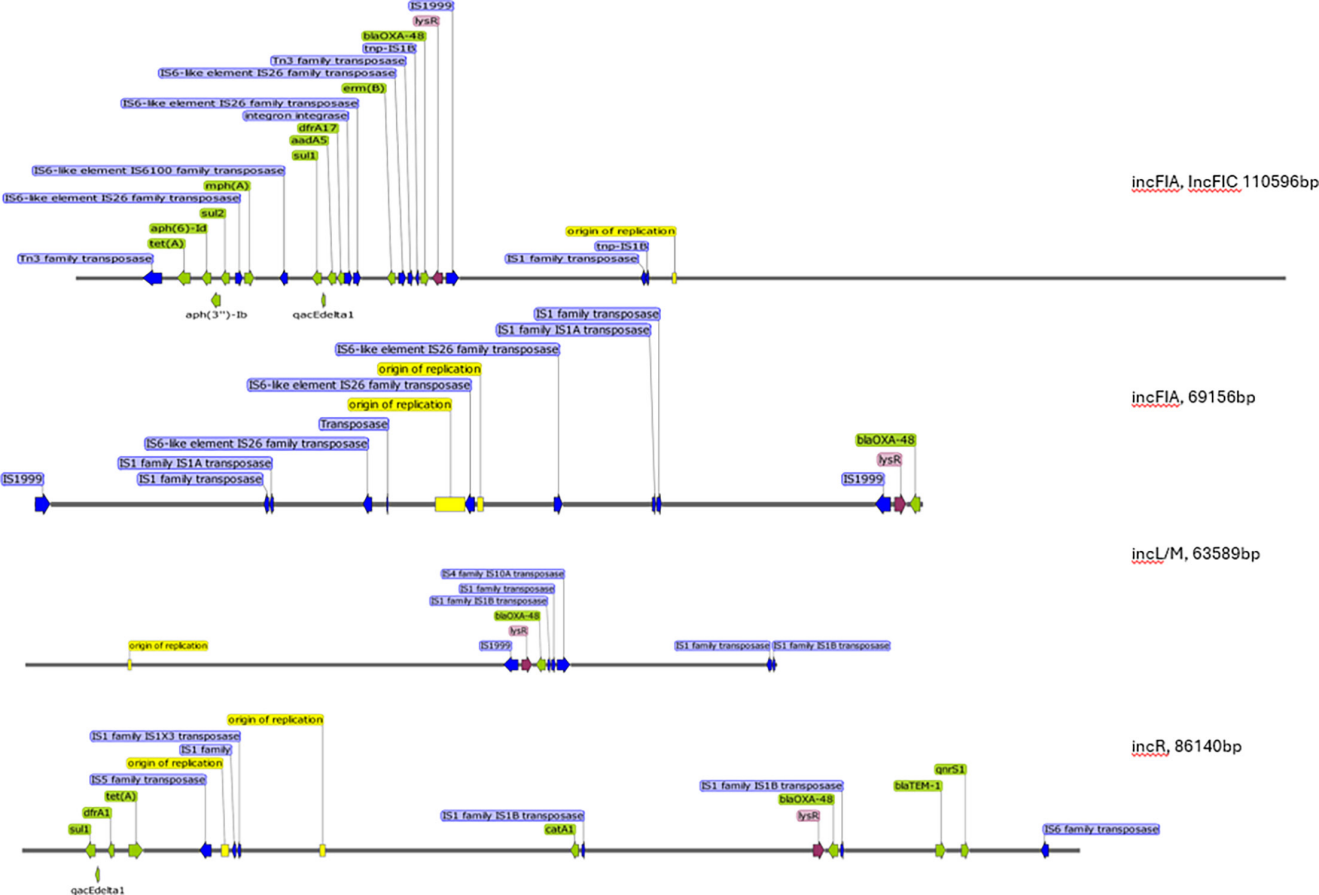
Layout of the different *bla*_OXA-48_ plasmids, based on replicon types. Only AMR genes, transposases, origin of replication and *LysR* genes were shown to improve readability.

Four main different replicon type plasmids were identified (Fig. 3): IncFIA+IncFIC, IncFIA, IncL/M (the main plasmid of this study, which includes mobstype LR025105), and IncR. The IncFIA+InCFIC encoded for, next to *bla*_OXA-48_ for *tet(A)*, *aph-Id*, *aph(3’’)-Ib*, *Sul2*, *mph(A)*, *sul1*, *qacEdelta1*, *aadA5*, *dfrA17* and *erm(B)*. *IncR* encoded for *sul1 dfrA1*, *tet(A)*, *catA1*, *bla*_TEM-1A_ and *qnrS1.* The IncL/M and incFIA replicon-type plasmids did not encode for any other resistance genes. All replicon-type plasmids encoded for a plethora of transposases such as IS1, IS6 and the *bla*_OXA-48_-associated IS1999.

### *bla*_OXA-48_ plasmid dynamics in a large hospital setting

In total, 12 different *bla*_OXA-48_ plasmid groups were identified belonging to four replicon types (Fig. 3, Table 2). Out of the 12 *bla*_OXA-48_ variant plasmid groups detected and classified by MASH nearest neighbour (distance=0.0001), three classified plasmid groups were found more than twice in our study population, classified as LN864820 (*n*=12), KX636096 (*n*=16) and LR025105 (*n*=38). Genetic diversity within each plasmid group was relatively small with, on average, two SNPs (max 59) for LR025105, four SNPs (max 29) for LN864820 and three SNPs (max 29) for KX636096. Given our main objective, we focused on the predominant plasmid (LR025105) that was part of the outbreak in 2011 (Fig. 1c). This plasmid group was the only plasmid type detected in the first 3 years of the study (2011–2013), with year 2014 marking the introduction of multiple other pOXA-48 MASH-classified plasmid types. Since 2014, plasmid group LR025105 was less frequently detected over the course of the study, particularly after 2016 where it was detected zero to two times per year at different hospital departments (Fig. 1c). Since 2014, 12 patients were carriers of plasmid group LR025105 (screening swabs were positive, and no clinical infections were detected). *Enterobacter hormaechei* (*n*=4), *C. freundii* (*n*=3), *K. pneumoniae* (*n*=3), *E. coli* (*n*=1) and *M. morganii* (*n*=1) were found. Eleven were known by the local infection prevention team (IPC), and one remained unclear. Based on these IPC historical records of these 11 patients, five were screened upon arrival due to hospitalization abroad and were found positive. Out of the remaining six patients, four were known carriers of *bla*_OXA-48_ *Enterobacterales*. Contact tracing investigations were therefore initiated for only two patients. These investigations did not find a link between any of the patients included in this study.

### Comparison of *bla*_OXA-48_ plasmids in context to Dutch and international plasmid populations

**Fig. 4.**
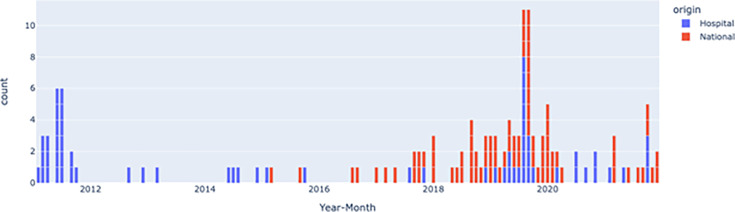
Frequency of plasmid LR025105 plasmids detected in this study (highlighted in blue) clustered with Dutch surveillance *bla*_OXA-48_ plasmid sequences (highlighted in red) over time. Structured national surveillance for CPE by short- and long-read sequencing started in 2016, as the incidence of CPE in the Netherlands before this hospital outbreak was negligible. It should be noted that a subselection was made for hospital isolates, due to capacity constraints to sequence all isolates encoding *bla*_OXA-48_, as explained in the methods section.

Based on the isolates submitted for national surveillance, MASH analysis (distance=0.0001) revealed that 35 distinct *bla*_OXA-48_ plasmid groups were found in the Netherlands from 2016 till 2021 compared to 12 in this study. Among the 346 (262 nationals and 84 hospitals) typed isolates, 39.8% (104) of the plasmid types were LR025105 compared to 17.5% (38) at our hospital. From a national perspective, from 2016 to 2021, each year 2, 8, 16, 17, 5 and 5 *K*. *pneumoniae* with a *bla*_OXA-48_ LR025105 plasmid were found, respectively (Fig. 4, Table S1, available in the online version of this article). These *K. pneumoniae* belonged to 29 different ST, of which ST395 was only found once, in 2018. For *E. coli*, 11 isolates were sent in for national surveillance, all with unique STs (Table S1), indicating the promiscuous nature of this plasmid. From 2018 to 2020, an increase in the incidence of LR025105 is seen nationally. In total, 55 isolates with an LR025105 pOXA-48 plasmid were sent in for national surveillance by other 29 hospitals in the Netherlands (Fig. 4). When reported, patients were hospitalized in either Morocco or Turkey, if they were recently hospitalized abroad. This has been described elsewhere in more detail [11].

## Discussion

In this study, we investigated the *bla*_OXA-48_ genetic diversity detected in a single hospital over the course of 11 years. Our results indicate that no persistence of the 2011 outbreak-associated plasmid was found and that the diversity of plasmids carrying *bla*_OXA-48_ resembles isolates collected by the Dutch national surveillance system. The European dissemination of a single *K. pneumoniae* clone carrying a *bla*_OXA-48_ gene embedded in transposon Tn1999.2 and located on a 62 kb IncL/M conjugative plasmid (categorized by MASH nearest neighbour as LR025105) caused a large outbreak when introduced in the Netherlands [5,6]. A review published in 2019 by Pitout *et al.* showed that only a few years later, this plasmid has become the most dominant pOXA-48 variant around the world [14]. The Dutch national surveillance data presented in this study confirms this was the most frequently detected *bla*_OXA-48_ plasmid type. At the hospital level, we see a decline of the 2011 outbreak-associated variant (MASH nearest neighbour; LR025105) being sporadically detected among the 84 *bla*_OXA-48_-positive isolates in the years that followed.

From 84 sequences, 12 different plasmid types carrying *bla*_OXA-48_ genes were identified in this study belonging to four different combinations of replicon types. These 12 plasmid types were also detected in the national surveillance system, suggesting that the sequenced isolates in this study form a reasonable representation of the national level. On a national level, the plasmid of initial concern, LR025105, was almost never found in the same ST twice, excluding the possibility of nationwide dissemination of specific bacterial clones. As so many different STs were observed carrying this specific plasmid, previous remarks regarding the promiscuous nature of this plasmid should be emphasized [14]. The CPE population in the hospital and the Netherlands was diverse after the outbreak with varying carbapenemase alleles and plasmid MOB types, suggesting multiple introductions into the country [8]. Additionally, epidemiological data back up that CPE carriage is associated with travel abroad [11].

One limitation of the study is sample selection. Given the large number of samples involved in the study, a selection of isolates had to be made due to financial constraints. Additionally, as highlighted in the introduction, the focus of this study was limited to the already known outbreak plasmid and did not investigate the transmission dynamics of the other plasmids found in this study. A follow-up study is planned to investigate these other *bla*_OXA-48_ plasmids in greater detail. To conclude, screening of 187,000 samples resulted in the detection of 374 *bla*_OXA-48_-positive patients in an 11-year study period. The 2011 outbreak-associated plasmid was only sporadically detected among the selected *bla*_OXA-48_-positive isolates, indicating that the outbreak was brought under control both on the organism as well as the plasmid level.

## supplementary material

10.1099/mgen.0.001335 Uncited Table S1.

## Abbreviations

Cfr: *Citrobacter freundii*
Cko: *Citrobacter koseri*
CPE: carbapenemase-producing *Enterobacterales*
Ecl: *Enterobacter cloacae* complex
Eco: *Escherichia coli*
ICU: intensive care unit
IPC: infection prevention team
Kpn: *Klebsiella pneumoniae*
Mmo: *Morganella morgani*
NGS: next-generation sequencing
